# Association between tooth loss and cognitive impairment in community-dwelling older Japanese adults: a 4-year prospective cohort study from the Ohasama study

**DOI:** 10.1186/s12903-018-0602-7

**Published:** 2018-08-20

**Authors:** Sho Saito, Takashi Ohi, Takahisa Murakami, Takamasa Komiyama, Yoshitada Miyoshi, Kosei Endo, Michihiro Satoh, Kei Asayama, Ryusuke Inoue, Masahiro Kikuya, Hirohito Metoki, Yutaka Imai, Takayoshi Ohkubo, Yoshinori Hattori

**Affiliations:** 1 Division of Aging and Geriatric Dentistry, Department of Oral Function and Morphology, Tohoku University Graduate School of Dentistry, 4-1 Seiryo-machi, Aoba-ku, Sendai, Miyagi 980-8575 Japan; 2 Japanese Red Cross Ishinomaki Hospital, Ishinomaki, Japan; 3 Division of Public Health, Hygiene and Epidemiology, Faculty of Medicine, Tohoku Medical and Pharmaceutical University, Sendai, Japan; 4 Department of Hygiene and Public Health, Teikyo University School of Medicine, Tokyo, Japan; 5 Department of Planning for Drug Development and Clinical Evaluation, Tohoku University Graduate School of Pharmaceutical Sciences, Sendai, Japan; 6 Department of Medical Informatics, Tohoku University Graduate School of Medicine, Sendai, Japan; 7 Department of Preventive Medicine and Epidemiology, Tohoku Medical Megabank Organization, Tohoku University, Sendai, Japan

**Keywords:** Cognitive impairment, Cohort study, Community-dwelling, Elderly, Tooth loss

## Abstract

**Background:**

Numerous prospective studies have investigated the association between the number of remaining teeth and dementia or cognitive decline. However, no agreement has emerged on the association between tooth loss and cognitive impairment, possibly due to past studies differing in target groups and methodologies. We aimed to investigate the association between tooth loss, as evaluated through clinical oral examinations, and the development of cognitive impairment in community-dwelling older adults while considering baseline cognitive function.

**Methods:**

This 4-year prospective cohort study followed 140 older adults (69.3% female) without cognitive impairment aged ≥65 years (mean age: 70.9 ± 4.3 years) living in the town of Ohasama, Iwate Prefecture, Japan. Cognitive function was evaluated with the Mini-Mental State Examination (MMSE) in baseline and follow-up surveys. Based on a baseline oral examination, the participants were divided into those with 0–9 teeth and those with ≥10 teeth. To investigate the association between tooth loss and cognitive impairment, we applied a multiple logistic regression analysis adjusted for age, sex, hypertension, diabetes, cerebrovascular/cardiovascular disease, hypercholesterolemia, depressive symptoms, body mass index, smoking status, drinking status, duration of education, and baseline MMSE score.

**Results:**

In the 4 years after the baseline survey, 27 participants (19.3%) developed cognitive impairment (i.e., MMSE scores of ≤24). Multiple logistic regression analysis indicated that participants with 0–9 teeth were more likely to develop cognitive impairment than those with ≥10 teeth were (odds ratio: 3.31; 95% confidence interval: 1.07–10.2). Age, male gender, and baseline MMSE scores were also significantly associated with cognitive impairment.

**Conclusions:**

Tooth loss was independently associated with the development of cognitive impairment within 4 years among community-dwelling older adults. This finding corroborates the hypothesis that tooth loss may be a predictor or risk factor for cognitive decline.

## Background

Dementia is the leading reason for older people needing long-term care in Japan. It is also a major social problem because the need for long-term care not only reduces the patient’s quality of life but also places psychological and financial burdens on the family and other caregivers. According to World Health Organization estimates, the number of people with dementia worldwide reached around 50 million in 2017 and is increasing by nearly 10 million new cases annually [1]. Because no effective treatment for dementia has been established, the modifiable predictors and risk factors must be identified in order to reduce the incidence of dementia.

Numerous prospective studies [2–13] have reported an association between oral health, particularly the number of remaining teeth, and dementia or cognitive decline in old age. Conversely, some reports [14–18] found no such association. Recent systematic reviews [19–21] have reached no consensus position on the question. The inconsistencies between studies may arise from differences in target groups and methodologies. Some studies have analyzed specific occupational groups or nursing home residents [3–5, 9, 12, 14]. Several studies have determined the number of remaining teeth through subject-administered questionnaires [7–10, 15, 18], whereas some others have used clinical oral examinations [2, 11, 13, 16, 17]. Furthermore, although baseline cognitive function is closely related to subsequent cognitive decline, only two studies [8, 11] have examined baseline cognitive function as a confounding factor.

We therefore aimed to elucidate the association between tooth loss, as evaluated through oral examinations, and the subsequent development of cognitive impairment in community-dwelling older adults while considering baseline cognitive function.

## Methods

### Study design and population

**Fig. 1:**
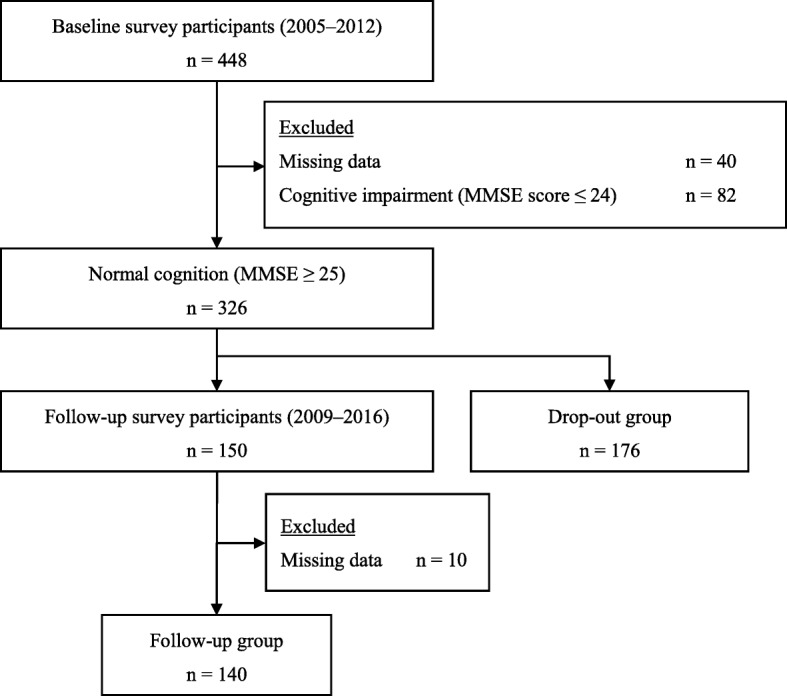
Flow diagram of study participants

This study was performed as a component of the Ohasama Study, a prospective cohort study on hypertension/cardiovascular disease that has followed general inhabitants of Ohasama, a rural town in northern Japan’s Iwate Prefecture, since 1986 [22, 23]. Figure 1 shows the study flow diagram. From 2005 to 2012, 448 inhabitants aged ≥65 years participated in the baseline survey. Of them, we excluded 40 participants with missing data and 82 participants who exhibited cognitive decline on cognitive function tests. The remaining 326 inhabitants were included as participants. Of them, 150 (46%) participated in the follow-up survey between 2009 and 2016. After excluding 10 participants with incomplete follow-up survey data, we included 140 participants in the final analysis as the “follow-up group.” The mean (± standard deviation) follow-up period was 4.0 ± 0.1 years. We included 176 people who did not participate in the follow-up survey in our “drop-out group.”

This study was approved by the Institutional Review Board of the Tohoku University Graduate School of Pharmaceutical Science and conformed to the principles of the Declaration of Helsinki. Written informed consent to participate was obtained from all participants.

### Measurements

Tooth loss at the time of the baseline survey was evaluated via count of the number of remaining teeth by specially trained dentists. The median number of remaining teeth was 10, so the participants were divided into those with 0–9 teeth and those with ≥10 teeth. We defined having 0–9 teeth as “multiple tooth loss.”

Cognitive function was evaluated with the Mini-Mental State Examination (MMSE) [24] in the baseline and follow-up surveys. Participants with a total score of ≤24 (full score: 30 points) were defined as having cognitive impairment. This cut-off reportedly achieves 83% sensitivity and 93% specificity in diagnosing dementia in Japanese patients [25]. We defined hypertension as a home blood pressure of ≥135/85 mmHg [26], use of antihypertensive medications, or a history of hypertension; diabetes mellitus as a non-fasting glucose concentration of ≥200 mg/dL [27], a glycated hemoglobin concentration of ≥6.5%, use of anti-diabetes medications, or a history of diabetes mellitus; cerebrovascular/cardiovascular disease as a history of atrial fibrillation, heart disease, or cerebrovascular disease; and hypercholesterolemia as a total cholesterol level of ≥220 mg/dL, use of anti-hypercholesterolemia medications, or a history of hypercholesterolemia. Depressive symptoms were evaluated with the Zung Self-Rating Depression Scale (SDS) [28]. The maximum SDS score is 80, and participants with scores of ≥40 were defined as having depressive symptoms. Body mass index (BMI) was calculated as weight in kilograms divided by height in meters squared. Participants were categorized as being current smokers/drinkers or not being current smokers/drinkers. Duration of education was categorized as being either less than 10 years or at least 10 years.

### Statistical analysis

Bivariate analyses were performed with Student’s *t*-test or the Wilcoxon rank sum test for continuous variables and the chi-squared test for categorical variables. A multiple logistic regression analysis was used to calculate adjusted odds ratios (ORs) and their 95% confidence intervals (CIs) for the development of cognitive impairment within 4 years of the baseline survey. Because few participants developed cognitive impairment, we applied logistic regression with penalized maximum likelihood estimation for rare events analysis [29]. The statistical significance threshold was *p* < 0.05 for all tests. Statistical analyses were performed with JMP Pro 12 (SAS Institute, Cary, NC, USA) and Stata 14 (StataCorp LLC, College Station, TX, USA).

## Results

The baseline characteristics of the follow-up and drop-out groups are shown in Table 1. The drop-out participants were significantly older and more likely to have hypertension than the follow-up group participants were, but the two groups did not significantly differ in sex, duration of education, BMIs, baseline MMSE scores, or frequencies of multiple tooth loss, current smoker status, current drinker status, diabetes mellitus, cerebrovascular/cardiovascular disease, hypercholesterolemia, or depressive symptoms. Of the follow-up participants, 27 (19.3%) had cognitive impairment at the follow-up survey. Table 2 compares the baseline characteristics of individuals who retained normal cognition and those who developed cognitive impairment by the follow-up survey. Compared to the participants with normal cognition, those with cognitive impairment were significantly older, had significantly lower baseline MMSE scores, and were significantly more likely to be male and to exhibit multiple tooth loss.

**Table 1:** Characteristics of the follow-up and drop-out groups

	Follow-up (*n* = 140)	Drop-out (*n* = 176)	*P*-value
Age (mean ± SD)	70.9 ± 4.3	73.2 ± 4.8	< 0.0001
Male (%)	30.7	29.0	0.7
Hypertension (%)	53.6	66.5	0.02
Diabetes (%)	11.4	17.6	0.1
Cerebrovascular/cardiovascular disease (%)	12.9	15.3	0.5
Hypercholesterolemia (%)	60.7	54.0	0.2
Depressive symptoms (%)	10.7	10.2	0.9
BMI (mean ± SD)	23.6 ± 3.2	23.9 ± 2.8	0.4
Current smoker (%)	7.1	5.7	0.6
Current drinker (%)	39.3	32.4	0.2
< 10 years of education (%)	73.6	76.7	0.5
Baseline MMSE score (mean ± SD)	27.7 ± 1.6	27.8 ± 1.5	0.4
Multiple tooth loss (%)	50.7	46.6	0.5

*P*-values were determined with Student’s *t*-test or the Wilcoxon rank sum test for continuous variables and the chi-squared test for categorical variables. Multiple tooth loss was defined as having 0–9 remaining teeth

*BMI* body mass index, *MMSE* Mini-Mental State Examination, *SD* standard deviation

**Table 2:** Baseline characteristics of individuals with normal cognition and those who developed cognitive impairment

	Normal cognition (*n* = 113)	Cognitive impairment (*n* = 27)	*P*-value
Age (mean ± SD)	70.5 ± 4.2	72.3 ± 4.5	0.04
Male (%)	26.6	48.2	0.03
Hypertension (%)	52.2	59.3	0.5
Diabetes (%)	9.7	18.5	0.2
Cerebrovascular/cardiovascular disease (%)	11.5	18.5	0.3
Hypercholesterolemia (%)	61.1	59.3	0.9
Depressive symptoms (%)	8.9	18.5	0.1
BMI (mean ± SD)	23.4 ± 3.0	24.3 ± 4.0	0.2
Current smoker (%)	7.1	7.4	0.9
Current drinker (%)	41.6	29.6	0.3
< 10 years of education (%)	71.7	81.5	0.3
Baseline MMSE score (mean ± SD)	28.0 ± 1.6	26.4 ± 1.2	< 0.0001
Multiple tooth loss (%)	46.0	70.4	0.02

*P*-values were determined with Student’s *t*-test or the Wilcoxon rank sum test for continuous variables and the chi-squared test for categorical variables. Multiple tooth loss was defined as having 0–9 remaining teeth

*BMI* body mass index, *MMSE* Mini-Mental State Examination, *SD* standard deviation

Table 3 shows the ORs and 95% CIs for developing cognitive impairment. In the age- and sex-adjusted model, multiple tooth loss was associated with an increased likelihood of developing cognitive impairment (OR: 3.39; 95% CI: 1.29–8.88). This association remained (OR: 3.31; 95% CI: 1.07–10.2) even after adjusting for age, sex, hypertension, diabetes, cerebrovascular/cardiovascular disease, hypercholesterolemia, depressive symptoms, BMI, smoking status, drinking status, duration of education, and baseline MMSE score. Greater ages, male gender, and lower baseline MMSE scores were also significantly associated with developing cognitive impairment. We did not find an interaction between age and tooth loss (*p* = 0.925).

**Table 3:** Multiple logistic regression model for development of cognitive impairment

	Age- and sex-adjusted model		Fully adjusted model^a^	
	OR (95% CI)	*P*-value	OR (95% CI)	*P*-value
Age	1.07 (0.98–1.18)	0.1	1.14 (1.01–1.29)	0.03
Male gender	3.34 (1.32–8.46)	0.01	4.60 (1.25–16.7)	0.02
Hypertension			1.28 (0.44–3.70)	0.6
Diabetes			3.77 (0.79–18.0)	0.1
Cerebrovascular/cardiovascular disease			2.62 (0.68–10.1)	0.2
Hypercholesterolemia			1.68 (0.56–5.00)	0.3
Depressive symptoms			2.50 (0.54–11.4)	0.2
BMI			1.04 (0.87–1.25)	0.6
Current smoker			0.48 (0.69–3.42)	0.5
Current drinker			0.50 (0.14–1.72)	0.3
Duration of education			1.31 (0.33–5.14)	0.7
Baseline MMSE score			0.48 (0.31–0.74)	0.001
Multiple tooth loss	3.39 (1.29–8.88)	0.013	3.31 (1.07–10.2)	0.037

The objective variable for the multiple logistic regression analysis was whether cognitive function declined within 4 years, and the explanatory variable was whether multiple tooth loss was present. Multiple tooth loss was defined as having 0–9 remaining teeth

^a^Adjusted for age, sex, hypertension, diabetes, cerebrovascular/cardiovascular disease, hypercholesterolemia, depressive symptoms, BMI, current smoker status, current drinker status, duration of education, and baseline MMSE score

*BMI* body mass index, *CI* confidence interval, *MMSE* Mini-Mental State Examination, *OR* odds ratio, *SD* standard deviation

## Discussion

In the present study, we examined whether multiple tooth loss, as evaluated with professional clinical oral examinations, is associated with developing cognitive impairment in community-dwelling older adults. We found that the presence of multiple tooth loss significantly increased the risk of developing cognitive impairment within 4 years independently of age, sex, hypertension, diabetes, cerebrovascular/cardiovascular disease, hypercholesterolemia, depressive symptoms, BMI, smoking status, drinking status, duration of education, and baseline MMSE score. This finding suggests that maintaining healthy dentition reduces the risk of cognitive impairment.

This study is one of the few longitudinal studies [2, 11, 13, 16, 17] to investigate the association between tooth loss, as evaluated with oral examinations, and cognitive impairment in community-dwelling older adults. A survey of community-dwelling older adults in South Korea [2] reported that a paucity of remaining teeth was related to the onset of dementia within 2.4 years. A 5-year prospective cohort study of Japanese community residents [11] reported that tooth loss predicted the development of mild memory impairment, and another 5-year cohort study of older adults in Japan [13] found that tooth loss was associated with an increased risk of developing all-cause dementia and Alzheimer’s disease. Our observation of a significant association between tooth loss and cognitive dysfunction corroborates these previous studies’ results. However, some studies [16, 17] found no association between tooth loss and cognitive decline. These studies differ from the others in that the participants were all older women [17] or in that the participants included middle-aged people as well as older ones [16].

In this study, we included the baseline MMSE score as a confounding factor in the multivariate analysis in order to consider the possible effect of mild cognitive decline that went undetected in screening tests. However, multiple tooth loss remained associated with cognitive impairment even after adjusting for baseline MMSE scores. Only two previous studies [8, 11] have adjusted for baseline cognitive function. A cohort study of older Japanese adults [8] found that having few teeth without dentures was associated with an increased risk of dementia independently of forgetfulness. A 5-year prospective cohort study of Japanese community residents [11] found that tooth loss was associated with developing mild memory impairment even after adjusting for baseline MMSE scores.

Several mechanisms may explain the association between tooth loss and cognitive impairment. First, the association may depend on chronic inflammation arising from periodontal disease, which is a major cause of tooth loss in later life. Previous studies showed that periodontal disease was the most common reason for tooth extraction in patients older than 45 years [30, 31]. Therefore, chronic exposure to periodontal disease–related inflammation is presumably a more serious problem in older adults with multiple tooth loss than in those without multiple tooth loss. Clinical attachment loss [32] and alveolar bone resorption [33], both of which indicate the cumulative history of periodontal disease, are associated with cognitive impairment. Systemic inflammatory reactions arising from periodontal disease are a hypothesized risk factor for Alzheimer’s disease [34–36].

Second, the association may depend on changes in food intake and nutritional status due to tooth loss. Poor nutritional status and nutrient deficiencies are reportedly associated with cognitive decline and Alzheimer’s disease [37–39]. A nationwide survey in the United States [40] found that individuals with fewer than 28 teeth had significantly lower intakes of carrots, tossed salads, and dietary fiber and had lower serum concentrations of β-carotene, folic acid, and vitamin C relative to individuals who were fully dentate. A survey of older Japanese adults [41] found that tooth loss was associated with decreased intakes of vegetables, fish, and shellfish. Therefore, macronutrient and micronutrient deficiencies resulting from decreased masticatory performance may contribute to cognitive impairment in older adults with missing teeth.

Third, the association may arise from deteriorations in masticatory performance reducing brain stimulation. Mastication-related trigeminal nerve sensory inputs reportedly increase cerebral blood flow [42], which in turn promotes arousal and activates a broad region that includes the sensory motor cortex, supplementary motor cortex, insular cortex, thalamus, and cerebellum [43]. Alzheimer’s disease model rats fed soft foods exhibit deficiencies in memory and learning abilities relative to rats fed hard foods [44], and rats with extracted molars exhibit reduced acetylcholine release in the cerebral cortex and deficiencies in spatial memory [45]. Therefore, masticatory dysfunction due to tooth loss may adversely affect brain function and thereby promote cognitive impairment.

Besides tooth loss, the factors significantly associated with cognitive impairment were advanced age, male gender, and low baseline MMSE scores. However, unlike tooth loss, these additional factors are unmodifiable. Practicing proper oral health to maintain healthy dentition may reduce the risk of future cognitive impairment. Furthermore, maintaining masticatory performance through prostheses and balanced nutritional intakes may reduce the negative impact of tooth loss on cognitive function. A 4-year prospective cohort study of individuals aged ≥65 years in Japan’s Aichi Prefecture [8] found that those with few teeth and no dentures had an increased risk of dementia relative to those with ≥20 teeth, but the risk of dementia was not significantly elevated in individuals with few teeth who used dentures. Intervention studies are needed to clarify whether restoring masticatory function and balancing nutritional intakes can prevent cognitive impairment.

This study has several limitations. First, the follow-up rate was not high (46%), and the follow-up pool might have been biased towards individuals who were healthier and had greater health awareness. Therefore, the possibility of selection bias should be considered when generalizing the present findings. However, this bias will not affect the internal validity of the relationship between tooth loss and the onset of cognitive impairment. Further, the follow-up and drop-out groups did not differ in any baseline characteristics except for age and hypertension rates. As for oral health, the participants’ average baseline number of teeth, measured from 2005 to 2012, was approximately five fewer than the average number in age-matched older Japanese adults recorded in national survey data from 2011 [46]. This suggests that our participants might not have been in good oral health. In addition, we may have lacked the participation of elderly people who were developing severe cognitive impairment during the 4 year follow-up; therefore, there is a possibility that the association between tooth loss and cognitive impairment was underestimated. Second, we did not evaluate dementia onset as an outcome. Instead, we screened for dementia with the MMSE, but this test is an indicator of cognitive function used worldwide in research and clinical practice. Its reliability for diagnosing dementia has been shown for Japanese patients [25]. Third, the timecourse of tooth loss was not taken into account. A previous prospective study showed that the rates of tooth loss and periodontal disease progression predicted subsequent cognitive decline [4]. Fourth, there is the problem of reverse causality. A previous study reported that cognitive decline was associated with increased odds of complete tooth loss, infrequent toothbrushing, and higher plaque levels [47]. Decline in cognitive function might increase the risk of tooth loss through poor oral hygiene because of reductions in oral health consciousness. In the present study, participants with cognitive decline at the baseline survey were excluded and the baseline MMSE scores were included as a confounding factor in the multivariate analyses to avoid the effect of reverse causality; however, there is the possibility that some participants who lost their teeth due to the influence of mild cognitive decline that was not detected in our evaluation were included. Finally, we could not adjust for all possible confounding factors. For example, low socioeconomic status, absence of spouse, unhealthy lifestyles, and certain genes are potentially associated with cognitive decline, but we could not collect data on these factors (although duration of education is a potential proxy for socioeconomic status). However, we did control for many potential confounders, including medical history, based on measurements from medical examinations, and our findings were robust.

## Conclusions

This prospective cohort study of community-dwelling older adults indicated that multiple tooth loss was associated with the development of cognitive impairment. This finding supports the hypothesis that tooth loss is a predictor or risk factor for cognitive decline. Both tooth loss and cognitive impairment are chronically accumulated over time. Further studies should be conducted to confirm the true nature of the impacts of tooth loss on cognitive impairment.

## Abbreviations

BMI: body mass index
CI: confidence interval
MMSE: Mini-Mental State Examination
OR: odds ratio
SDS: Zung Self-Rating Depression Scale
